# Alternating hyperthyroidism and hypothyroidism in Graves’ disease

**DOI:** 10.1002/ccr3.1700

**Published:** 2018-07-09

**Authors:** Mimi Wong, Warrick J. Inder

**Affiliations:** ^1^ Department of Diabetes and Endocrinology Princess Alexandra Hospital Brisbane QLD Australia; ^2^ Faculty of Medicine The University of Queensland Brisbane QLD Australia

**Keywords:** Graves’ disease, hyperthyroidism, hypothyroidism

## Abstract

Spontaneously oscillating thyroid function in Graves’ disease is a rare phenomenon. Switching between TSH receptor stimulating antibodies (TSAb) and TSH receptor blocking antibodies (TBAb) most likely accounts for presentations of alternating hyperthyroidism and hypothyroidism. To achieve stability of thyroid function, definitive therapy is recommended to remove the pathological thyroid.

## 1 INTRODUCTION

Graves’ disease (GD) is an autoimmune thyroid disease usually associated with hyperthyroidism. There have been cases of patients switching from hyperthyroidism to hypothyroidism, and even rarer patients flipping from hypothyroidism to hyperthyroidism.^1^ However, a case of spontaneously alternating hyperthyroidism and hypothyroidism in Graves’ disease is comparably an even rarer phenomenon. It is thought that the switching of stimulating TSH receptor antibodies (TSAb) and blocking TSH receptor antibodies (TBAb) has a role in alternating thyroid function. We present a case of spontaneously oscillating thyroid function over a 15‐year period.

## 2 CASE HISTORY

A 37‐year‐old Caucasian female, who apart from a history of GD had no significant medical history, was referred to the Endocrine Clinic in 2016. At the time of her initial diagnosis of GD in 2002, she was hyperthyroid with symptoms of fatigue and heat intolerance (Table 1). She was initially managed at another institution with carbimazole for approximately 2 years, which resulted in disease control. Following this period she was in remission until in 2007, then became spontaneously hypothyroid. She was subsequently commenced on thyroid hormone replacement, consisting of a combination of thyroxine and thyroid extract. While on thyroid hormone replacement she was symptomatically well with normal thyroid function tests (TFTs). In May 2015, she developed recurrence of hyperthyroidism with palpitations, tremor, anxiety, increased appetite, and heat intolerance. Her thyroid hormone replacement was changed from an unconventional form, 50‐mg thyroid extract daily in combination with alternating 50‐μg and 100‐μg thyroxine, to T3 alone in July 2015. At this point the dose of her total thyroid hormone replacement was also reduced. Her symptomatic hyperthyroidism persisted, and subsequently her thyroid hormone replacement was ceased. She was commenced on 10‐mg carbimazole twice a day in December 2015, prior to referral to our care.

**Table 1:** Change in thyroid function tests over time

Year	Free T4 (10‐20 pmol/L)	Free T3 (2.5‐6.8 pmol/L)	TSH (0.4‐4.0 mU/L)	TRAb (<1 IU/L)	TSI (active Graves’ >0.55 U/L)	TPO Ab (<35 U/mL)	Graves’ Treatment
2002	25	11.8	<0.05	35		1100	Managed with carbimazole for 2 y
2007	10	2.8	100	3.2		6100	Commenced on thyroid hormone replacement. From 2011 she was on 50‐mg thyroid extract & alternating 50‐μg and 100‐μg thyroxine
July 2015	29	12.2	<0.05	8			Thyroid hormone replacement changed to T3. Initially changed to 10 μg in the morning & 5 μg at night, then to 15 μg in the morning and 10 μg at night
Aug 2015	27	12.1	<0.05			537	
Sep 2015	23	9.1	<0.05				
Oct 2015	21	9.6	<0.05				
Jan 2016	31	11	<0.05				One 10‐mg carbimazole twice a day
Mar 2016	16	7.0	<0.05	7.3		102	Carbimazole reduced to 10 mg daily, though had variable compliance
Apr 2016	19	7.4	<0.05				
Sept 2016	16	6.3	<0.05	1.9			Self‐cessation of carbimazole in June 2016
Oct 2016	12	5.7	<0.05	2.4			
Dec 2016	12	3.6	0.3	2.1			
Feb 2017	16	4.4	0.3		3.00		
Apr 2017	16	4.7	0.2		4.80		

TSH, thyroid stimulating hormone; TRAb, TSH receptor antibody; TSI, thyroid stimulating immunoglobulin; TPO, thyroid peroxidise antibody.

On presentation to our institution in March 2016, she reported symptomatic improvement on carbimazole. On examination, her hands were warm and moist with fine tremors. Her thyroid was not enlarged, with no associated bruit. Cardiovascular, neurological and orbital examinations were otherwise unremarkable. There were no signs of Graves’ ophthalmopathy.

## 3 DIFFERENTIAL DIAGNOSIS, INVESTIGATIONS, AND TREATMENT

**Figure 1:**
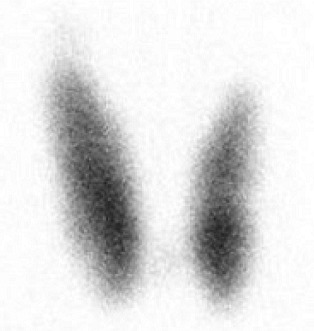
Radionuclide (Technetium‐Labeled) Thyroid Scan Consistent with Graves’ Disease

Given the unusual history of oscillating hyperthyroidism and hypothyroidism, a radionuclide Tc99 m thyroid scan was arranged to distinguish between thyroiditis and GD (Figure 1). The scan showed increased uptake bilaterally, with total uptake of 6.1% (reference range: 0.2%‐2.2%) consistent with active GD, with no retrosternal extension. TSH receptor antibody (TRAb) was positive at 7.3 IU/L (reference range: <1.0). Thyroid function tests showed a free T4 of 16 pmol/L (reference range 10‐20), free T3 of 7.0 pmol/L (reference range 2.5‐6.8) and TSH of <0.05 mU/L (reference range 0.4‐4.0).

Carbimazole was reduced to 10 mg once daily. Although she had been advised to continue taking her antithyroid medication, she stopped the carbimazole herself in June 2016. Following this her TFTs have almost normalized, with normal free T4 and free T3 and TSH just below the reference range.

## 4 OUTCOME AND FOLLOW‐UP

As her TFTs spontaneously improved, she has remained off carbimazole. However given the unpredictable nature of her GD, definitive treatment such as thyroidectomy or radioactive iodine has been offered, although she remains reluctant to take up either of these options. In addition, the issue of medical therapy of GD during pregnancy has also been discussed. However, she currently has expressed no plans for a future pregnancy.

## 5 DISCUSSION

This case highlights the challenges of managing a patient with GD and spontaneously oscillating thyroid function, particularly given her reluctance to undergo definitive therapy.

The switching of hyperthyroidism to hypothyroidism is uncommon in GD and most episodes occur several years after the cessation of antithyroid medications.^2^ Comparably, the flipping of patients from hypothyroidism to hyperthyroidism is much rarer, with only 37 such cases reported in the literature by 2014.^1^ The presentation of alternating hyperthyroidism and hypothyroidism therefore is an even rarer phenomenon in GD, and is described in very few case reports.^3^, ^4^ A possible reason for why it is uncommonly encountered in clinical practice may be because many patients with GD are referred relatively early in their disease course for definitive treatment, such as total thyroidectomy or radioactive iodine, thus eliminating the possibility for spontaneous oscillation of thyroid function.

Previously, it was thought that people with GD only had stimulating TSH receptor antibodies. However, now it is increasingly recognized that both TSAb and TBAb can be produced concurrently in the same patient^5^; the presence of hyperthyroidism or hypothyroid may depend on the balance between stimulating and blocking antibodies.^6^ The phenomenon of TSH receptor antibody switching between stimulating and blocking is infrequent.^4^ In a large study by Takasu & Matsushita, who followed‐up patients over 10 years, found that among 34 hypothyroid patients with TBAb predominance, only two subjects later developed TSAb‐dominant hyperthyroidism. Out of 98 TSAb predominant hyperthyroidism, only two subjects later developed TBAb‐dominant hypothyroidism.^7^ In a recent publication where TSAb and TBAb were quantified with a reporter gene bioassay using Chinese hamster ovary cells, <1% (10/1079) patients with autoimmune thyroid disease which included Hashimoto's thyroiditis and GD, were positive for both TSAb and TBAb.^5^

Unfortunately, we do not have the availability of TBAb measurement at our institution and only recently have been able to quantify TSAb. Monitoring of TRAb is important in pregnant women with GD, as TSAb and TBAb can cause neonatal thyrotoxicosis and hypothyroidism, respectively, and affect neonatal development.^8^ Thyroid peroxidase antibodies (TPO Ab), which are associated with Hashimoto's thyroiditis, can exist in patients with GD. Although TPO Ab were not frequently requested in our case, the titer of TPO Ab increased during the hypothyroid phase. Hashimoto's thyroiditis following Graves’ hyperthyroidism can occur, due to the expansion of autoantibody generation from TSH receptor initially to TPO subsequently,^9^ and change in balance between TSAb and TBAb.

There have been several theories put forth to account for the switching of TSAb and TBAb. Rarely, treatment with levothyroxine may increase thyroid autoantibody production including TSAb, such that hypothyroid patients can later become hyperthyroid.^4^ It is hypothesized that elevated thyroid hormone, through thyroxine replacement, affects the immune system such that it inhibits T regulatory cells and enhances expression of costimulatory molecules by dendritic cells, which are both important in antibody production and TSAb secretion.^4^ Treatment with antithyroid medications such as carbimazole reduces thyroid autoimmunity and TSAb secretion,^10^ and following treatment the balance could switch to predominately TBAb.

Autoimmune thyroid disease and oscillating thyroid function, similar to this case report, can occur following treatment with alemtuzumab for multiple sclerosis.^11^ Gilbert et al^11^ suggest that the switching of thyroid states following treatment with alemtuzumab was also likely due to change in balance between TSAb and TBAb. In addition, switching of thyroid autoantibodies has been found to occur more frequently in certain demographic groups including women, patients aged between 39 and 44 and those of Japanese background.^4^

An unconventional form of thyroid hormone replacement therapy, namely thyroid extract, was used in this case. Although it contains iodine, this is unlikely to have contributed to the flipping of hypothyroidism to hyperthyroidism in this case. Teng et al^12^ found that iodine supplementation in their study did not increase the incidence of GD or hyperthyroidism. Rather they found that high iodine intake was associated with autoimmune thyroiditis causing hypothyroidism.

Due to the unpredictable nature of the disease in patients with alternating hyperthyroidism and hypothyroidism, definitive therapy would be recommended to stabilize their thyroid hormone replacement in the long term. A block and replace regimen may improve stability over the short to intermediate term. Definitive therapy includes both thyroidectomy and radioactive iodine, which has the disadvantage in that it can only be performed in the hyperthyroid phase. Without definitive treatment, management can be challenging, and close monitoring of the patient along with regular thyroid function test will be required for ongoing follow‐up.^13^, ^14^

## CONFLICT OF INTEREST

None declared.

## AUTHORSHIP

MW: saw the patient in the Endocrinology outpatient clinic, gained consent for publication of the case report, collated the clinical data and investigation results and wrote the first draft of the manuscript. WJI: was the supervising consultant at the Endocrinology outpatient clinic, followed up the patient, reviewed, and edited the manuscript.
